# Oligometastatic Breast Cancer With Cutaneous Involvement and Paraneoplastic Hypercalcemia in a Young Woman

**DOI:** 10.7759/cureus.100498

**Published:** 2025-12-31

**Authors:** Marta Viana-Pereira, Cristiana H Martins, Marta Almeida

**Affiliations:** 1 Medical Oncology, Braga Local Health Unit (ULSB), Braga, PRT; 2 Life and Health Sciences Research Institute (ICVS) School of Medicine, University of Minho, Braga, PRT

**Keywords:** breast carcinoma with cutaneous involvement, multimodal therapy, neuroendocrine differentiation, oligometastatic breast cancer, paraneoplastic hypercalcemia

## Abstract

Cutaneous involvement in breast cancer is typically associated with locally advanced or neglected disease. In young women, such presentations are often associated with a more aggressive biological behavior.

We report the case of a woman in her early 40s presenting with a large, cutaneous-involved, hormone receptor-positive, human epidermal growth factor receptor 2 (HER2)-negative, right breast cancer with neuroendocrine differentiation. Imaging revealed axillary, supraclavicular, and right cervical lymph node involvement, with no evidence of distant metastasis. Laboratory evaluation at diagnosis demonstrated malignant hypercalcemia refractory to medical treatment. The patient received neoadjuvant chemotherapy, achieving resolution of hypercalcemia and a significant clinical and radiologic response, followed by surgery, radiotherapy, and adjuvant endocrine therapy. At 24 months post-surgery, the patient remains free of disease.

This case underscores the importance of prompt multidisciplinary management in aggressive presentations of breast cancer, even when associated with paraneoplastic syndromes.

## Introduction

Breast cancer is the most diagnosed malignancy in women worldwide and a leading cause of cancer-related death, particularly in women under 45 years of age, being also a significant source of morbidity in this young and active population [1,2]. In younger women, breast cancer diagnosis is often delayed due to the absence of routine screening and low awareness [2,3]. While cutaneous involvement is rare in early-stage breast cancer disease, its presence often implies advanced or aggressive tumors, especially in younger patients, whereas in older ages, it is more frequently associated with neglected diseases [4].

Neuroendocrine differentiation of breast cancers is poorly understood but may carry prognostic implications [5-7]. These tumors are often hormone receptor-positive and may exhibit aggressive behavior [5-7].

Malignant hypercalcemia is an uncommon but serious and potentially life-threatening paraneoplastic syndrome, usually associated with late-stage cancer, and requires immediate treatment, particularly in higher serum calcium concentrations and symptomatic patients [8]. It is also associated with poorer patient prognosis, and, in some cases, its resolution only occurs with effective oncological control [8].

We report this case due to its unusual and aggressive presentation, characterized by rapid tumor growth, extensive cutaneous involvement, neuroendocrine differentiation, and paraneoplastic hypercalcemia at diagnosis in a young woman. These features highlight the importance of recognizing adverse tumor biology beyond stage alone and underscore the role of early multidisciplinary and curative-intent treatment in selected patients.

## Case presentation

**Figure 1:**
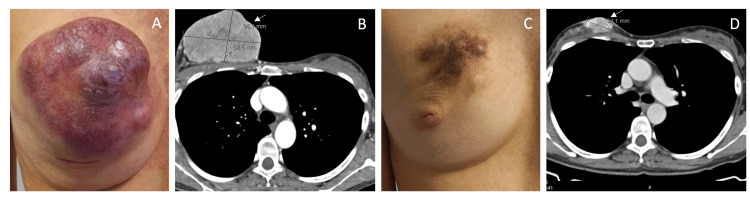
Right breast clinical and imaging findings, before and after neoadjuvant chemotherapy (A) Observation at diagnosis showing a violaceous and infiltrative mass occupying the upper quadrants of the right breast, consistent with cutaneous involvement. (B) Contrast-enhanced CT scan of the chest demonstrating a large right breast lesion measuring approximately 95 mm in its greatest dimension before treatment (arrow). (C) Marked clinical improvement after neoadjuvant chemotherapy (five months), with residual post-treatment pigmentation and skin retraction. (D) Post-chemotherapy CT scan showing a significant reduction of the primary lesion to approximately 34 mm (arrow).

A woman in her early 40s, with no significant past medical history, presented with a five-month history of a progressively enlarging right breast mass. On physical examination, a violaceous, hard, and irregular mass occupied the upper quadrants of the right breast (Figure 1A), accompanied by ipsilateral axillary (~3 cm) and supraclavicular (~2 cm) lymphadenopathy.

Laboratory work-up revealed asymptomatic malignant hypercalcemia with a total calcium level of 14.2 mg/dL, corrected to serum albumin (Table 1). The condition was initially managed with intravenous fluids and bisphosphonate, achieving only a partial biochemical response followed by relapse.

**Table 1:** Relevant laboratory findings at diagnosis CA 15-3: cancer antigen 15-3; CEA: carcinoembryonic antigen; PTH: parathyroid hormone

Parameter	Result	Reference range	Units
Calcium	15.6	8.3-10.6	mg/dL
Albumin	5.1	3.4-5.0	g/dL
Albumin-corrected calcium	14.2	8.3-10.6	mg/dL
Creatinine	0.7	0.6-1.10	mg/dL
Phosphorus	2.5	2.4-5.1	mg/dL
Alkaline phosphatase	85	46-116	U/L
PTH	<4.3	18.5-88.0	pg/mL
Magnesium	23	15-26	mg/L
CA 15.3	66.8	<32.4	U/mL
CEA	57.8	0.0-5.0	ng/mL

Core needle biopsy confirmed an invasive carcinoma of no special type (NST) exhibiting neuroendocrine differentiation, Nottingham grade 2 [9,10]. Immunohistochemistry demonstrated strong estrogen and progesterone receptor positivity (>90%), absence of human epidermal growth factor receptor 2 (HER2) expression (score 0), and a Ki-67 proliferation index of 82% [10]. Biopsy of the axillary lymph node confirmed metastatic carcinoma consistent with breast origin.

Imaging studies, including breast MRI, mammography, ultrasound, contrast-enhanced thoraco-abdominopelvic CT, fluorodeoxyglucose-positron emission tomography (FDG-PET), and bone scintigraphy, revealed a large malignant mass in the right breast measuring 95 mm in its greatest dimension (Figure 1B). There was ipsilateral axillary, supraclavicular, and latero-cervical lymph node involvement, but no evidence of further distant metastases (cT4bN3cM1; stage IV) [11]. Germline genetic testing identified a heterozygous BRIP1 gene variant (c.617C>T), classified as a variant of uncertain clinical significance.

Following multidisciplinary tumor board discussion, the case was classified as oligometastatic, and neoadjuvant chemotherapy was initiated with curative intent [5-7,12,13].

The patient received four cycles of dose-dense doxorubicin and cyclophosphamide followed by 12 weekly doses of paclitaxel. A partial clinical and radiologic response was observed, with the reduction of the primary tumor to 23 mm (Figure 1C, 1D). Hypercalcemia resolved completely after the second chemotherapy cycle, supporting its paraneoplastic etiology [8].

The patient subsequently underwent a right modified radical mastectomy. Histopathological examination revealed a multifocal invasive carcinoma NST, grade 2, showing partial pathological response to chemotherapy (Miller-Payne grade 3) [10,14]. Metastatic involvement was identified in 11 of 14 dissected lymph nodes, corresponding to ypT3N3aR0 [11].

Following surgery, the patient received adjuvant radiotherapy, including coverage of the supraclavicular and cervical lymphatic regions, concomitantly with endocrine therapy initiated with a luteinizing hormone-releasing hormone (LHRH) analogue in combination with anastrozol. After finishing radiotherapy, ribociclib, a cyclin-dependent kinase inhibitor (iCDK4/6), and bisphosphonate were introduced as part of adjuvant systemic treatment, maintaining the previously initiated endocrine therapy. At 24 months after surgery, the patient remains clinically and radiologically disease-free.

## Discussion

This case illustrates several key aspects relevant to clinical practice. Cutaneous involvement in breast cancer, particularly in younger women, is uncommon and often signals an aggressive biology [3,4,15]. In this patient, clinical and imaging findings were most consistent with direct tumor extension to the overlying skin, rather than distant cutaneous metastasis. The absence of routine screening for women under 45 years, combined with low disease awareness, may contribute to delayed diagnosis and poorer outcomes in this population.

Neuroendocrine differentiation, though rare, is recognized in a small subset (2-5%) of breast carcinomas and may carry prognostic significance. These tumors often express hormone receptors but may exhibit variable clinical behavior, particularly when associated with a high Ki-67 index, as observed in our patient [5-7,16]. These tumors may express neuroendocrine markers (chromogranin, synaptophysin) and tend to occur in hormone receptor-positive contexts, though clinical implications remain under study [5,7,16]. Currently, there is limited evidence to support alternative chemotherapy regimens, such as platinum-based therapy, in the absence of small cell morphology or definitive neuroendocrine carcinoma features. In this case, standard anthracycline- and taxane-based neoadjuvant chemotherapy was selected in accordance with guidelines for high-risk hormone receptor-positive breast cancer, achieving both tumor response and metabolic control.

Paraneoplastic hypercalcemia is most commonly mediated by humoral mechanisms through the tumor secretion of parathyroid hormone-related peptide (PTHrP), which mimics parathyroid hormone (PTH) activity and leads to increased bone resorption and renal calcium reabsorption. This mechanism may occur even in the absence of bone metastases. In this case, the suppressed PTH level, lack of bone involvement, and rapid normalization of calcium after systemic chemotherapy strongly support a humoral mechanism. This underscores the importance of prompt tumor control in the management of oncologic metabolic emergencies [8,17].

The aggressive and locally advanced presentation observed in this case likely reflects adverse biological characteristics rather than tumor burden alone. Despite hormone receptor positivity, the tumor exhibited a markedly high proliferative index (Ki-67 82%), extensive cutaneous involvement, and neuroendocrine differentiation, all features associated with more aggressive clinical behavior. Recognition of this biological profile has important implications for management, favoring early systemic therapy, a multimodal curative-intent approach, and intensified adjuvant treatment strategies aimed at reducing the risk of recurrence. Moreover, oligometastatic breast cancer is increasingly viewed as a potentially curable condition, and aggressive multimodal treatment may offer survival benefits in carefully selected patients [12,13]. The favorable outcome observed in our patient supports this strategy and highlights the value of individualized, multidisciplinary care in complex and biologically aggressive breast cancer presentations.

## Conclusions

Despite presenting with aggressive clinical features, including cutaneous involvement, neuroendocrine differentiation, and paraneoplastic hypercalcemia, this patient with oligometastatic breast cancer remains disease-free at two years after surgery. Early initiation of systemic therapy, combined with comprehensive multimodal management, was crucial to achieving durable disease control.

This case highlights the importance of prompt recognition and multidisciplinary management of paraneoplastic manifestations in breast cancer. The favorable outcome supports the curative-intent approach in selected oligometastatic cases and underscores the potential reversibility of paraneoplastic syndromes with effective oncologic control.
